# Template-Free and Surfactant-Free Synthesis of Selective Multi-Oxide-Coated Ag Nanowires Enabling Tunable Surface Plasmon Resonance

**DOI:** 10.3390/nano10101949

**Published:** 2020-09-30

**Authors:** Chi-Hang Tsai, Shih-Yun Chen, Alexandre Gloter, Jenn-Ming Song

**Affiliations:** 1 Department of Materials Science and Engineering, National Taiwan University of Science and Technology, Taipei 106, Taiwan; bar_tsai@outlook.com; 2 Laboratoire de Physique des Solides, Université Paris-Saclay, 91405 Orsay, France; alexandre.gloter@u-psud.fr; 3 Department of Materials Science and Engineering, National Chung Hsing University, Taichung 402, Taiwan; 4 Research Center for Sustainable Energy and Nanotechnology, National Chung Hsing University, Taichung 402, Taiwan; 5 Innovation and Development Center of Sustainable Agriculture, National Chung Hsing University, Taichung 402, Taiwan

**Keywords:** oxide shell, Ag nanowire, core-shell structure, surface plasma resonance, refractive index

## Abstract

Without using templates, seeds and surfactants, this study successfully prepared multi-oxide-layer coated Ag nanowires that enable tunable surface plasmon resonance without size or shape changes. A spontaneously grown ultra-thin titania layer onto the Ag nanowire surface causes a shift in surface plasmon resonance towards low energy (high wavelength) and also acts as a preferential site for the subsequent deposition of various oxides, e.g., TiO_2_ and CeO_2_. The difference in refractive indices results in further plasmonic resonance shifts. This verifies that the surface plasma resonance wavelength of one-dimensional nanostructures can be adjusted using refractive indices and shell oxide thickness design.

## 1. Introduction

Metal-oxide core-shell nanowires have emerged to improve the functionality and applicability of metal nanowires. For instance, Ag@TiO_2_ nanowires show many advantages when used as the photoelectrodes for dye-sensitized solar cells [1,2,3,4], e.g., the prevention of electron-hole recombination and anti-corrosion from electrolytes. Moreover, the surface plasmon resonance of core-shell nanostructures encourages the enhanced localized electromagnetic field, which provides light absorption improvements by dye molecules and thus, a better harvesting efficiency than traditional TiO_2_ electrodes.

CeO_2_ is also a major oxide shell material. Zhang et al. [5] reported that for Ag@CeO_2_ nanospheres the oxygen storage capability of the CeO_2_ shell significantly improves the catalytic properties and reduces the conversion temperature for CO oxidation, which can be ascribed to oxygen vacancies generation to provide abundant absorption sites for oxygen species due to the interfacial interactions between the Ag core and CeO_2_ shell. 

With respect to synthetic one-dimensional metal-oxide core-shell nanostructure production methods, Dong et al. [6] used hydrolysis with post-annealing to obtain an anatase TiO_2_ shell on Ag nanowires. However, the Ag core could be damaged or even disrupted. Eom et al. proposed a sophisticated and complicated process [7], including the application of a nanoimprint and evaporation techniques to fabricate Ag nanowires onto polymer templates. This was followed by TiO_2_ electrodeposition onto the Ag nanowire surface. Most metal/CeO_2_ core-shell structures are successfully synthesized in the form of nanoparticles, but only a few researchers have developed core-shell nanowires. One relevant project was conducted by Galicia et al. [8], who applied the sol-gel precipitation method to produce Ag@CeO_2_ nanotubes with Kirkendall voids by leveraging a faster CeO_2_ shell diffusion rate than that of the Ag core. The majority of core-shell nanowires synthesized using the aforementioned methods come with amorphous shells. An annealing process is needed to improve oxide crystallization.

Core-shell nanowires with a metal core and dual-oxide shells comprising native oxides are the only ones that have been successfully synthesized, e.g., Cu/Cu_2_O/CuO or Cu/Cu_2_O/ZnO [9,10]. Our group successfully synthesized template-free and surfactant-free Ag nanowires with a uniform spontaneous ultra-thin titania shell (~0.5 nm) on TiO_2_ coated Si substrate [11]. The titania was denoted as TiO_2−*x*_ due to a deficiency of oxygen. Using these ultra-thin TiO_2−*x*_ coated Ag wires as the template, this study proposes a facile method to prepare metal-cored nanowires with multi-oxide shells. Electron energy loss spectroscopy (EELS) and UV-visible spectroscopy were conducted to explore the influence of oxide shell variation on the Ag nanowire plasmon resonance properties.

## 2. Experimental Procedures

**Figure 1:**
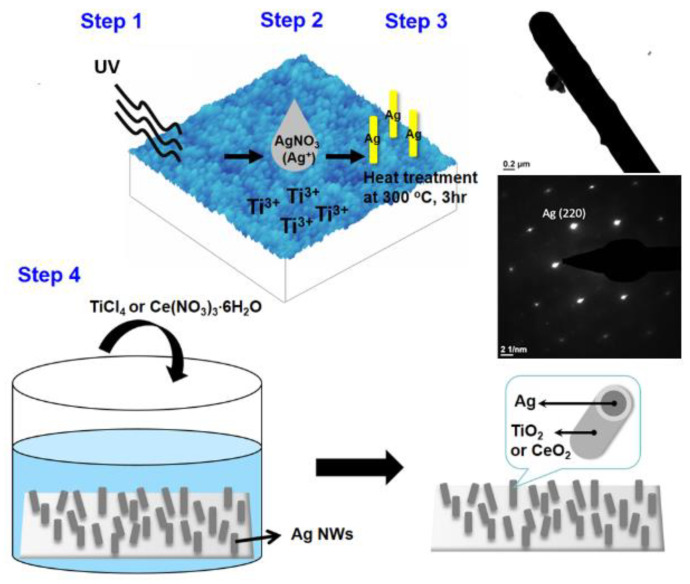
Synthesis process schematic illustration of the steps for obtaining Ag nanowires with multi-oxide-shells, including step 1. UV irradiation on TiO_2_ substrate, step 2. Dropping AgNO_3_ solution on TiO_2_ substrate, step 3. Heat treatment for forming Ag nanowires, and step 4. Coating of outer oxide shells, as well as the TEM analytical results of the synthesized Ag nanowires (TEM image and selected-area diffraction pattern).

Figure 1 illustrates the workflows used to synthesize Ag-cored nanowires with multi-oxide shells. TiO_2_ thin films were prepared via the sol gel method by dipping Si wafers into the gels and then were spun at 1000 rpm for 30 s. The TiO_2_ solutions consisted of isopropylalcohol (IPA)/titanium isopropoxide (TTIP)/hydrogen chloride (HCl) with a volume ratio of 170:12:0.4, which were aged at room temperature (20 °C) for 2 days before dipping. The coated samples were annealed at 500 °C in an oxygen atmosphere for 8 h to achieve better crystallinity.

Fifteen µL of 0.05 M silver nitrate (I) (AgNO_3_) solution was dropped onto the TiO_2_ substrate, and then subjected to proper UV exposure (Step 1 and 2 in Figure 1) [12]. A heat treatment was conducted at 300 °C for 3 h followed by furnace-cooling (Step 3). The parameters of the heat treatment for nanowire synthesis were determined based on our previous investigations [12,13]. The TEM image and selected area diffraction pattern given in Figure 1 verify that the obtained Ag nanowires were single crystalline growing towards (220). The average wire diameter ranged from 100~150 nm, and the average length was 8.3 µm. In step 4, the synthesized Ag@TiO_2−*x*_ nanowires served as templates and were dipped into 0.05 M titanium tetrachloride (TiCl_4_) or ammonium ceric nitrate (Ce(NO_3_)_3_·6H_2_O) aqueous solutions. Reactions were held at 200 °C for 4 h.

A GIXRD (grazing incidence X-ray diffraction, Bruker D8 Discover SSS, Billerica, MA, USA) was used to analyze the crystalline phase (at the parameters Cu kα (40 kV 100 mA); λ = 0.15418 nm; incidence angle = 2θ; scan range = 20°–60°; scan speed = 2° per minute). Using small incident angles for the incoming X-ray beam, the diffractions can be made surface-sensitively, and are suitable for detecting the oxide shells on Ag nanowires. A scanning electron microscope (JEOL JSM-6500F, Tokyo, Japan) and transmission electron microscopy (Philips Tecnai G2 and Nion USTEM200, Kirkland, WA, USA) with an acceleration voltage set at 200 kV were used for microstructural observation. Elemental line scanning and mapping were performed using EELS, which was equipped in a Nion USTEM200 operated at either 100 or 200 keV. The low loss spectra were acquired with an energy dispersion of typically 20 meV per channel, millisecond acquisition time and an energy resolution of 0.3 eV. A UV-Vis spectrophotometer (Jasco V-670, Easton, MD, USA) was also used to measure absorption peaks using surface plasmon resonance.

## 3. Results and Discussion

**Figure 2:**
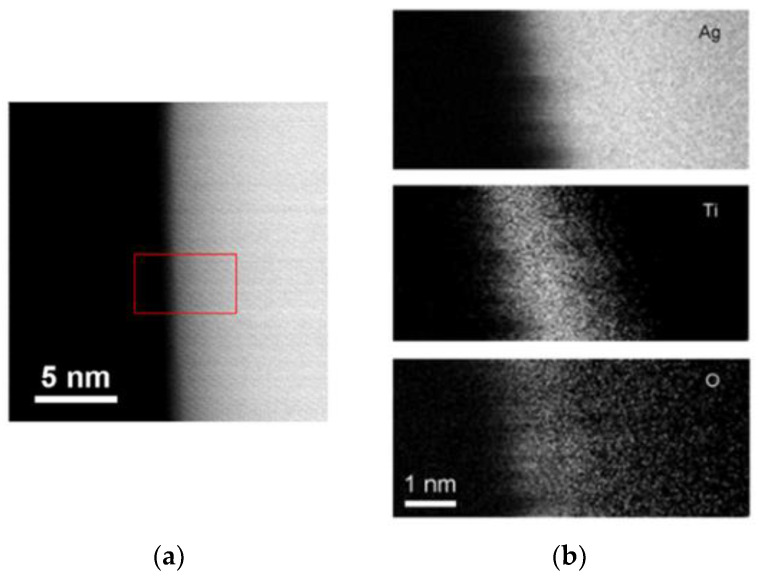
(**a**) high-angle annular dark-field (HAADF)-STEM image and (**b**) EELS mapping of Ag NW edge grown onto the TiO_2_ substrate after heating at 300 °C for 3 h. The elemental mapping was slightly shifted due to specimen drift.

**Figure 3:**
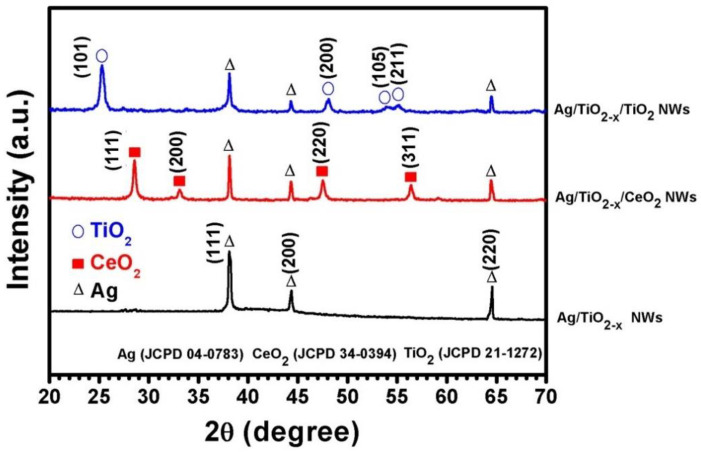
GIXRD patterns of the Ag/TiO_2−*x*_, Ag/TiO_2−*x*_/TiO_2_ and Ag/TiO_2−*x*_/CeO_2_ NWs (The shell thicknesses were 1 nm for TiO_2−*x*_, 5–7 nm for TiO_2_, and 4–6 nm for CeO_2_).

Figure 2 presents the HAADF (high-angle annular dark-field) image and EELS mapping results for Ag nanowires, indicating an ultra-thin oxide layer with the thickness of around 1 nm that uniformly covered the whole wire surface. Our previous study [11,13] revealed that this ultra-thin oxide spontaneously forms during nanowire growth. The spontaneously grown thin oxide layer is oxygen deficient and the Ti valence is between Ti^4+^ and Ti^3+^. Therefore, the Ag nanowires could be denoted using Ag/TiO_2−*x*_. Figure 3 shows the XRD diffraction pattern of Ag nanowires (Ag/TiO_2−*x*_) and those coated with TiO_2_ and CeO_2_ (Ag/TiO_2−*x*_/ TiO_2_ and Ag/TiO_2−*x*_/CeO_2_). For Ag/TiO_2-x_ samples, only diffraction peaks of Ag were detected. In addition to the Ag diffraction peaks, Ag/TiO_2−*x*_/TiO_2_ nanowire samples also reveal anatase-TiO_2_ signals, while Ag/TiO_2−*x*_/CeO_2_ nanowires show both the Ag and CeO_2_ diffraction peaks.

**Figure 4:**
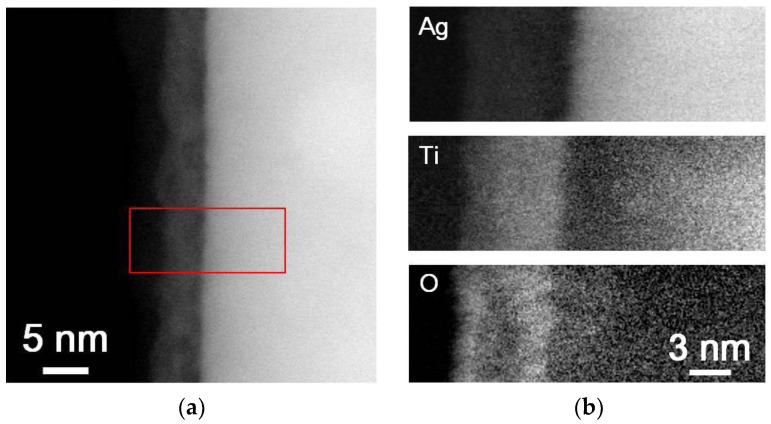

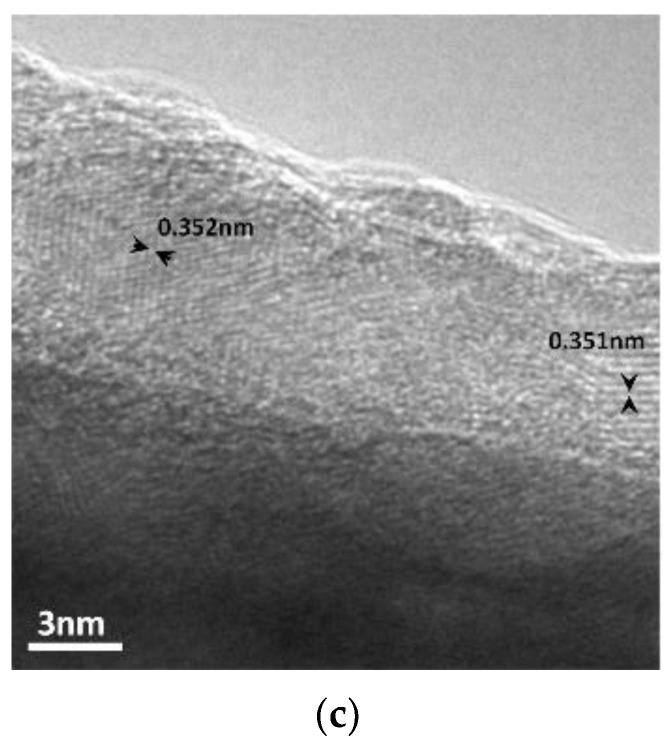
Microstructure analytic results of the outmost oxide shell of a Ag/TiO_2−*x*_/TiO_2_ nanowire: (**a**) STEM-HAADF image, (**b**) EELS elemental mapping taken from the marked region in (**a**), (**c**) HRTEM images.

**Figure 5:**
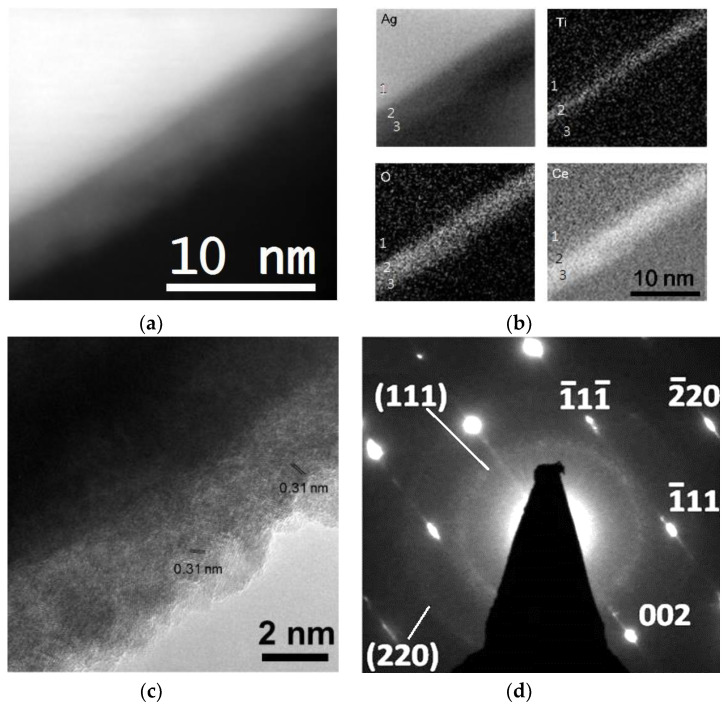
Ag/TiO_2−*x*_/CeO_2_ nanowire oxide shell microstructure analytical results: (**a**) HAADF image, (**b**) EELS elemental mapping taken from the marked region in (**a**) (point 1, point 2 and point 3 indicate Ag wire, TiO_2−*x*_ and CeO_2_, respectively), (**c**) HRTEM images, and (**d**) selected area diffraction patterns.

Figure 4 presents the microstructural analytical results for Ag/TiO_2−*x*_/TiO_2_ nanowires. STEM-HAADF and EELS elemental mapping images (Figure 4a,b) display a continuous second phase enriched with Ti and O formed on the Ag nanowires surface with a layer thickness of about 5–7 nm. The lattice spacing of (101) planes, 0.35 nm (Figure 4c), again identifies the outmost oxide shell was anatase TiO_2_. STEM-HAADF images and corresponding EELS elemental mapping, respectively, shown in Figure 5a,b clearly show the dual shell feature of Ag/TiO_2−*x*_/CeO_2_ nanowires, i.e., Ti-rich inner shell and Ce-rich outer shell. The thicknesses of inner and outer shells were about 1 nm and 4–6 nm, respectively. The lattice spacing, 0.31 nm shown in Figure 5c, is consistent with that of CeO_2_ (111) planes. The selected area diffraction patterns, Figure 5d, reveal spots from crystalline Ag nanowire and rings from CeO_2_ nanocrystals. TiO_2−*x*_ between the Ag wire and CeO_2_ was too thin to be observed in Figure 5d.

Unlike previous studies, the multi-oxide shell developed in our case is comprised of TiO_2−*x*_, TiO_2_, and CeO_2_, which are other than native oxides of metal cores (e.g., copper oxides to copper). This can be referred to as the spontaneously formed oxide shell, TiO_2−*x*_, which serves as a buffer layer enhancing subsequent oxide adsorption and deposition, and thus facilitates the formation of multi-oxide shell metal-cored nanowires. It has been proposed that the TiO_2−*x*_ shell forms through Ti oxidation which is repelled from single-crystalline Ag during nanowire crystallization, and Ti is from TiO_2_ substrate dissolution in molten salt [11]. Ag@CeO_2−*x*_ nanowires can also be prepared using the same spontaneous growth method [14], thereby CeO_2−*x*_ can be developed as an alternative for the inner oxide shell.

**Figure 6:**
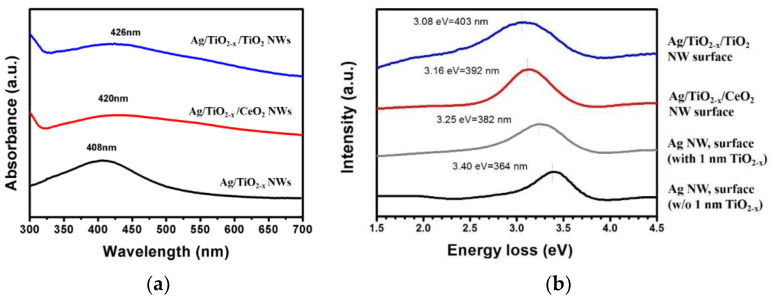
Nanowire optical properties: (**a**) UV-visible absorption spectra, and (**b**) low loss region of EELS.

Figure 6a illustrates the UV-Vis spectra of Ag@TiO_2-x_ nanowires and those with TiO_2_ or CeO_2_ outer oxide shells. Ag@TiO_2−*x*_ shows an absorption peak at 408 nm. It is worth noting that further coating with a second oxide shell, TiO_2_ or CeO_2_, gave rise to a red shift in the absorption peaks to 426 nm (Ag/TiO_2−*x*_/TiO_2_) and 420 nm (Ag/TiO_2−*x*_/CeO_2_). The low-loss EELS region (Figure 6b) indicates Ag nanowires without any oxide shell and surfactants exhibited a localized surface plasmon resonance (LSPR) at 3.40 eV (364 nm) [15,16]. SPR can be red-shifted due to spontaneously-grown ultra-thin oxide shell (inner shell) as well as an additional oxide layer (outer shell). As illustrated, LSPR appeared at 3.25 eV (382 nm) for Ag/TiO_2−*x*_ nanowires. A thicker extra oxide layer resulted in a greater shift, i.e., 3.16 eV (392 nm) for Ag/TiO_2−*x*_/CeO_2_ nanowires and 3.08 eV (403 nm) for Ag/TiO_2−*x*_/TiO_2_. The above results imply that the LSPR location can be tuned by the number of layers and oxide shell material selection, as well as the thickness control.

Studies on Ag-TiO_2_ and Ag-Al_2_O_3_ core-shell clusters [17,18] suggest that when the outer oxide layer refractive indices are greater than those of the inner metal, surface plasmon resonance shifts will be generated. The incident beam polarization affects the refracted beam. That is to say, a refracted beam is damped or attenuated. Its amplitude decreases as a function of the penetration depth. The higher the embedding medium refractive index, the greater the incident beam will be the polarized, which will weaken the restoration forces inside the polarized particles. This gives rise to the excitation of another oscillation mode resulting in resonance shifting towards the higher wavelength side or lower energy side.

With respect to the oxide shells in our cases, the TiO_2_ refractive index is 2.54 [19], while that of CeO_2_ is 2.2 [19]. The fact that TiO_2_ has a higher refractive index than CeO_2_ explains the greater red shift in TiO_2_-covered nanowires compared to Ag/TiO_2−*x*_/CeO_2_ nanowires.

## 4. Conclusions

A simultaneously-grown ultra-thin oxide on the Ag nanowire surface leverages the buffering characteristics for developing multi-oxide shell metal-cored nanowires. The EELS mapping demonstrates that an ultra-thinTiO_2−*x*_ layer with a thickness of about 1 nm fully covers Ag nanowires. Further deposition of similar or dissimilar oxides can proceed readily. The spectra results at the low-loss peak region verify surface plasmon peak resonance variation. The covering of one spontaneous TiO_2−*x*_ layer at approximately 1 nm on the Ag nanowire surface causes the shift in surface plasmon resonance toward low energy (high wavelength). Additional outer oxide shells magnify the shift. The LSPR shift can be controlled through the oxide refractive index selection. This study sheds light on the possibility for one-dimensional nanostructure preparations that enable tunable surface plasmon resonance without shape changes.
